# Feeding Habits of *Dentex maroccanus* and the Effect of Body Size

**DOI:** 10.3390/ani13050939

**Published:** 2023-03-05

**Authors:** Amalia Mina, Chryssi Mytilineou, Alexandros Kaminas, Anthi Rekleiti, Apostolos Siapatis, Aikaterini Anastasopoulou

**Affiliations:** 1 Hellenic Centre for Marine Research, Institute of Marine Biological Resources and Inland Waters, 19013 Anavyssos, Greece; 2 Department of Marine Sciences, University of the Aegean, 81100 Mytilene, Greece; 3 Department of Ichthyology and Aquatic Environment (DIAE), School of Agricultural Sciences, University of Thessaly, 38446 Volos, Greece

**Keywords:** *Dentex maroccanus*, diet ecology, feeding strategy, size effect, trophic level, Aegean Sea, Mediterranean Sea

## Abstract

**Simple Summary:**

Understanding the biology and ecology of Morocco dentex (*Dentex maroccanus*) requires knowledge of several life aspects, including its feeding habits. The findings of this study demonstrated that this species is a predator with a carnivorous character, feeding mainly on decapods. Gastropods, squids, clams, and fish are also included in the diet of Morocco dentex in the South Aegean Sea. The body size is an important factor affecting the diet of the species with the smaller individuals feeding more on small-sized clams and gastropods than the larger ones, which feed more on large-sized worms, fish, and mantis prawns. This research may contribute to a deeper understanding of the species’ feeding habits, which is important information for environmentally friendly fisheries management.

**Abstract:**

The feeding habits of organisms are important elements in their ecological role and are affected by several factors. The present study provides for the first time information on the diet and feeding strategy of *Dentex maroccanus* (Valenciennes, 1830) and examines the effects of various factors on the species’ feeding activity. Various indices (vacuity index, numerical and weight proportion, frequency of occurrence, alimentary coefficient, index of relative importance, diet breadth and overlap, Shannon–Wiener index, and trophic level) were estimated. The diet of the species consisted of 18 different prey taxa. The most important prey taxon was Decapoda. The study of the feeding strategy showed the narrow width of the species. Body size was found to significantly affect the species’ feeding habits. Polychaeta and Stomatopoda were found only in individuals with size ≥165 mm, Bivalvia mainly in sizes ≤120 mm, and Decapoda in the intermediate sizes. The largest individuals showed the lowest overlap with all other size groups. The trophic level increased from 3.7 in young individuals to 4.0 in larger sizes, indicating the carnivorous character of the species. The results of the present work contribute to a better knowledge of the species’ feeding ecology.

## 1. Introduction

Stomach content analysis constitutes an essential component for understanding, exploring, and contrasting trophic relationships between organisms, as well as population and community dynamics [1]. These studies are essential and fundamental in Marine Ecology and Fisheries Dynamics, providing useful information for fisheries management and ecosystem status assessment [2]. Several factors may affect the diet of the species, such as season, sex, and body size [3,4].

The Morocco dentex, *Dentex maroccanus* Valenciennes, 1830, is a ray-finned fish species of the family Sparidae. It is distributed in the Eastern Atlantic, from the Bay of Biscay to the Gulf of Guinea, sometimes even further north, and in the Southern and Eastern Mediterranean; it is not recorded in the Adriatic and Black Sea [5]. It is a demersal species [6] and may be found in a range of bottom types, mostly gravels or conglomerates [7]. It is mainly an inshore species, found in the Mediterranean at depths of 20 to 250 m [5] with the highest abundance between 50–70 m in depth [8]. However, recent studies have revealed that the species’ bathymetric distribution extends to 316 m in depth [9]. The distribution of the species was found to be related to environmental factors such as depth, temperature, and season [8,9,10]. It is a by-catch species of low economic value mostly fished by trawl and artisanal fisheries [11].

Studies on various biological aspects of *D. maroccanus* such as age, growth, length–weight relationship, reproduction, fecundity, and morphometry have been conducted in the Mediterranean to date [12,13,14,15,16,17,18,19,20,21,22]. Only one study on the diet of the species in the Atlantic Ocean has been published [23]. Two relevant studies in the Mediterranean are also known [24,25], while no information is available for the species in the Greek waters.

The objective of the present study is to investigate *D. maroccanus*’ diet and feeding strategy, and the effect of various factors (body size, season, sex) on its feeding activity. Diet overlap, breadth, and trophic level were also studied. The case study was conducted in the South Aegean Sea. The aim was to provide new information on the species’ diet in the study area and improve existing knowledge of the species’ feeding ecology.

## 2. Materials and Methods

**Figure 1:**
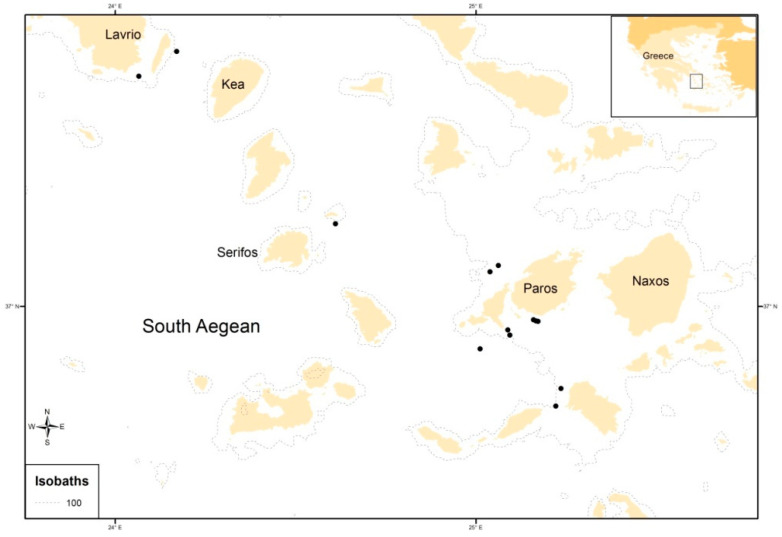
Sampling sites (as black spots) of *Dentex maroccanus* in the South Aegean Sea.

Sampling was carried out with a commercial bottom trawl in the South Aegean Sea (E. Mediterranean) (Figure 1) in September 2014 and May 2015, during the daytime and within the framework of the national project EPILEXIS. The sampling scheme included 84 hauls in a wider geographical area; however, a few specimens of the species occurred in most of them. Therefore, the samples for this study were taken from the hauls where the species was more abundant. A total of 416 specimens were collected at depths between 68 and 255 m. Fish were frozen promptly after capture and dissected in the laboratory, where the total weight (TW) (to the closest 0.001 g), the total length (TL) to the closest mm, the sex, and the maturity stage were recorded. TL ranged from 92 to 233 mm.

Stomachs were extracted and sectioned, and their content was weighted. A stereomicroscope was used to examine the content of the stomachs in order to identify prey items, which were counted and weighted. The intestinal contents were also extracted and examined qualitatively, but they were not taken into account in further analyses. Prey items were identified to the lowest feasible taxonomic level, depending on their digestion condition. However, most of the prey items in the stomachs were particles of the entire organism (e.g., otoliths, eyes or flesh of Cephalopoda, Decapoda appendages, parts of Gastropoda, and Bivalvia shells) or digested to the point that did not permit the identification to the species or genus level. When the organism was intact or a specific characteristic of an organism was found, the identification of the species level was successful.

The vacuity index (VI) was estimated as the proportion of empty stomachs. Stomach fullness was determined using two methods: (i) the repletion index (%RI) = stomach content weight × 100/body weight and (ii) a fullness empirical scale (with 0 representing empty stomach and 5 representing full stomach).

The study of the diet composition of the species was based on the following indices proposed by Hyslop [26]: numerical proportion (%N), frequency of occurrence (%F), and weight proportion (%W). Two additional indices were used to determine the importance of each prey in the diet of *D. maroccanus:* (i) the alimentary coefficient Q (Q = %F × %W) [26]; prey was divided into three groups based on the Q coefficient [27]: principal for Q > 200, secondary for 20 < Q < 200, and accidental for Q < 20 and (ii) the index of relative importance (IRI) of Pinkas et al. [28], modified by Hacunda [29]: IRI = (%N + %W) × %F. This index was calculated as %IRI = (IRI/Σ IRI) × 100, where Σ IRI was the sum of all IRI values.

The feeding strategy was determined using the method of Amundsen et al. [30], which plots prey-specifying abundance (p_i_) against the frequency of occurrence (%F). Expressed as a percentage, prey-specific abundance is a given prey taxon proportion in relation to all prey items observed in only those predators’ stomachs that contained the given prey taxon: p_i_ = 100 × ∑ S_i_ × ∑ S_ti_^−1^, where ∑ S_i_ is the sum of the stomachs comprising prey i, ∑ S_ti_ is the sum of all prey items found in only those predator stomachs that contained prey i.

In order to identify the effects of various factors on the feeding habits of *D. maroccanus*, permutational multivariate analysis of variance (PERMANOVA) was performed based on the Bray–Curtis similarity resemblance matrix for the prey abundance (N) using three factors: sex (male/female), season (summer/autumn), body size (juvenile/adult), and their interactions. The discrimination of the size groups was based on Mohdeb and Kara [16] findings that estimated the species’ first maturity at 144 mm TL. Distance-based redundancy analysis (db–RDA) was also used to demonstrate the resemblance of the prey items in the different identified groups.

Based on the results db–RDA, the largest individuals showed differentiation in their diet compared to the other individuals. Therefore, ontogenetic changes in the diet of *D. maroccanus* were further examined for the following 4 size groups (≤120 mm, 121–142 mm, 143–164 mm, ≥165 mm). Thus, 2 groups of juveniles and 2 groups of adults were created. In our samples, there were many prey taxa with zero values or values equal to 1. For this reason, the mean abundance and biomass of each prey taxon were considered for each one of the above-mentioned size groups. Based on these data, a cluster analysis was performed based on the Bray–Curtis similarity resemblance matrix of the average prey taxon abundance. Shade plots were produced to show the relationship between the cluster of size groups and prey taxa abundance. SIMPER analysis was used to identify the contribution of each prey taxon in differentiating the four examined size groups. The diversity in prey items of the four size groups was also examined using the Shannon–Wiener index (H′). All the above-mentioned analyses were implemented using the Primer v7 software package [31].

Diet overlap between the defined size groups of *D. maroccanus* was determined using Schoener’s formula: C_xy_ = 1 − 0.5 (∑ |px_i_ − py_i_|). C_xy_ is the dietary overlap, px_i_ is the proportion of prey taxon i (i terms of the relative abundance N) in the diet of group x, and py_i_ is the proportion of prey taxon i in the diet of group y. The values of this index range from 0 to 1, with 1 indicating complete overlap and 0 indicating no overlap. A value of index ≥0.6 was considered to indicate high overlap.

Diet breadth B for the size group i was calculated in this work for *D. maroccanus* using Levins index [32]: B = (n − 1)^−1^ [(∑_j_ p^2^_ij_)^−1^] − 1, where p_ij_ is the proportion of the diet of size group i on prey j and n is the number of prey taxa. The index values vary from 0 (the species consumes a simple item) to 1 (the species consumes different prey in equal proportion). Values of B ≥ 0.6 are considered high, between 0.4–0.6 moderate and below 0.4 low [33].

The trophic level (TrL), which expresses the position of organisms within the food webs, was calculated for each size group according to Pauly and Christensen [34] formula:

$${\mathrm{TrL}}_{i}=1+\overset{G}{\underset{j=1}{\sum }}\;\left({\mathrm{DC}}_{\mathrm{ij}}\times {\mathrm{TrL}}_{j}\right)$$

where TrL_j_ is the fractional trophic level of prey j, ${\mathrm{DC}}_{\mathrm{ij}}$ represents the proportion (%W) of prey j in the diet of specimen i, and G is the total number of prey species. Trophic levels of most fishes take values between 2 and 5 [35]. The trophic level values of each specimen were calculated using TrophLab routine [36].

## 3. Results

**Figure 2:**
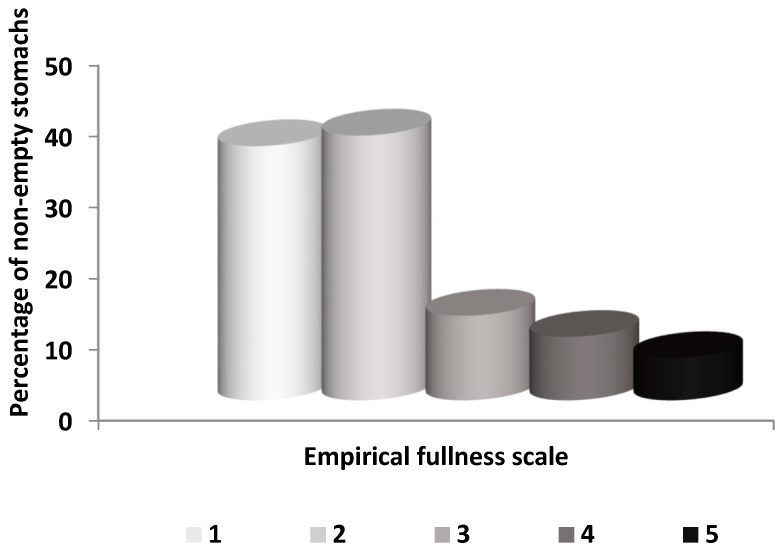
Percentage of the empirical fullness scale categories of non-empty stomachs (fullness scale with increasing order from 1 to 5).

Out of 416 *D. maroccanus* examined, 349 stomachs were empty (VI = 83.89%). Only 67 stomachs contained food remains. The size of the latter individuals ranged from 99 to 186 mm TL. There was no indication of regurgitation. The fullness index of non-empty stomachs based on RI was 0.74% (±0.1). From the stomachs with food, the empirical fullness scale of the stomachs showed a clear dominance of categories 1 and 2 (Figure 2).

Stomach content analysis of the 67 individuals revealed a total of 125 prey items, from 18 taxa (Table 1), weighing a total of 22.98 g. The examination of the intestinal content showed the following additional prey taxa: Algae, Foraminifera, Nematode, Echinodermata (Echinoidea), Gastropoda (Calliostomatidae, Cerithiidae, Drilliidae, *Homalopoma sanguineum*, Trochidae), Bivalvia (Myidae), and Crustacea (Amphipoda, Galatheidae, *Nephrops norvegicus*, Thalassinidae). Μicroplastics were also found in the intestine of some individuals.

The quantitative diet analysis of stomachs revealed that Decapoda unidentified was the most abundant prey identified in the stomachs of *D. maroccanus* (%N = 34.4), followed by Gastropoda (%N = 20.0). The heaviest prey taxon was also Decapoda unidentified (%W = 35.0), followed by Cephalopoda (%W = 18.9). In terms of frequency, Decapoda unidentified predominated followed by Gastropoda. The alimentary coefficient Q index showed that the primary prey in the diet of *D. maroccanus* was Decapoda unidentified, while benthic Decapoda, Gastropoda, Cephalopoda, Dendrobranchiata, Teleostei, and Bivalvia were identified as secondary prey. All the other taxa were classified as accidental prey. Similar results were also derived by the analysis of %IRI (Table 1).

**Figure 3:**
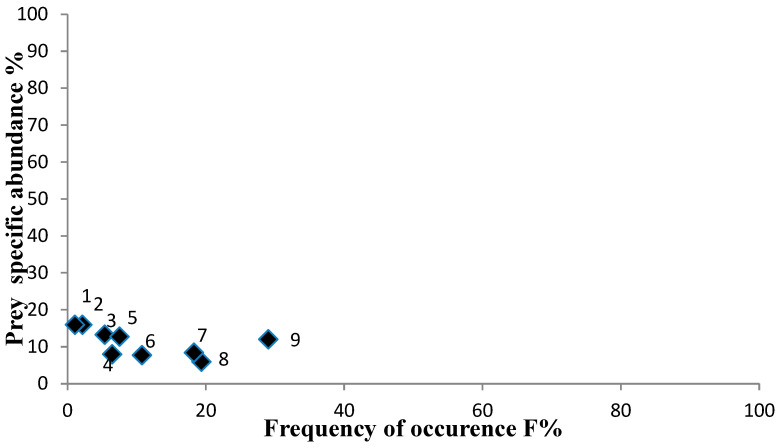
Feeding strategy plot of *Dentex maroccanus*. Prey types (as squares) are: 1. Polychaeta; 2. *Rissoides desmaresti*; 3. Teleostei; 4. Cephalopoda; 5. Dendrobranchiata; 6. Bivalvia; 7. Benthic Decapoda; 8. Gastropoda; 9. Decapoda unidentified.

The feeding strategy (Figure 3) showed that most prey taxa were located on the left side of the diagram with low prey-specific abundances (<20%) and relatively low frequency of occurrence (<40%), indicating that most prey taxa were rare in the diet of the species. Benthic Decapoda, Decapoda unidentified, and Gastropoda, which were more frequent, were located below the prey importance axis (50%), showing a kind of generalized feeding behavior towards these prey taxa.

PERMANOVA analysis showed that among the examined factors of body size, sex, and season, the only statistically significant factor affecting the *D. maroccanus* diet was body size (Table 2).

**Figure 4:**
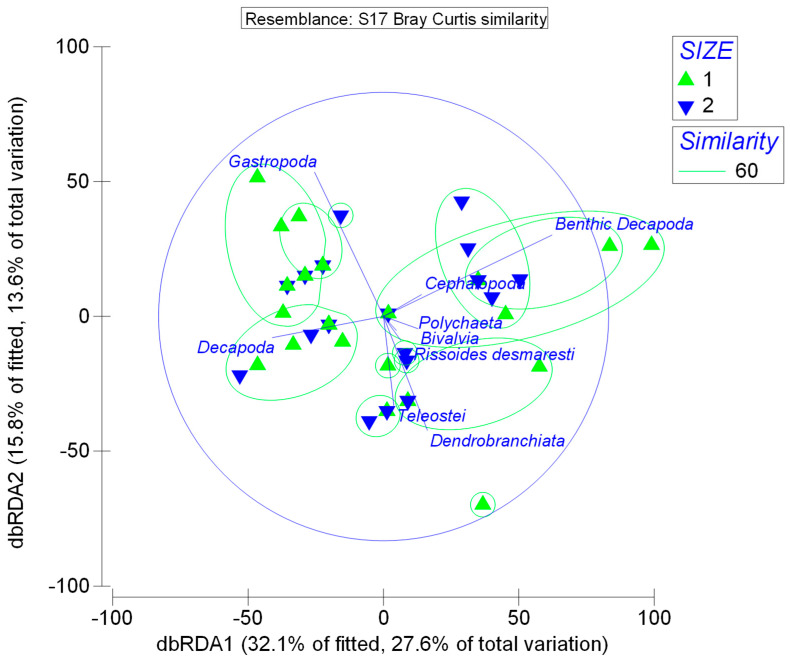
Distance based Redundancy Analysis (db−RDA plot). Size 1 = juvenile size group, Size 2 = adult size group.

The results of db–RDA analysis, although not very clear for the juveniles and adults of *D. maroccanus*, showed that the largest of the individuals of the size group of adults determined by Teleostei, *Rissoides desmaresti,* and Polychaeta were grouped separately than the other examined individuals (Figure 4).

**Figure 5:**
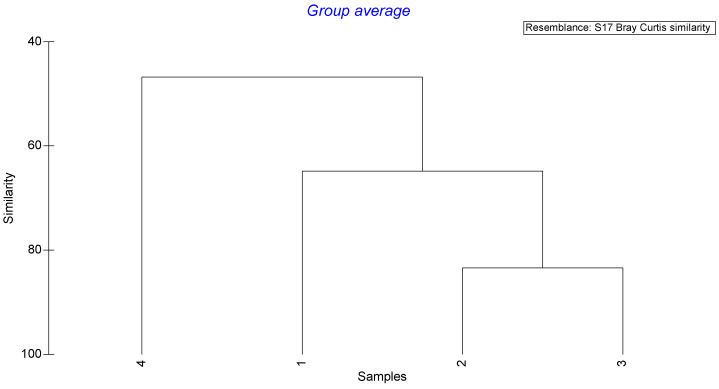
Dendrogram of four clusters of body sizes based on the Bray–Curtis distance. Size group 1: smaller juveniles (≤120 mm), group 2: larger juveniles (121–142 mm), group 3: smaller adults (143–164 mm), and group 4: larger adults (≥165 mm).

**Figure 6:**
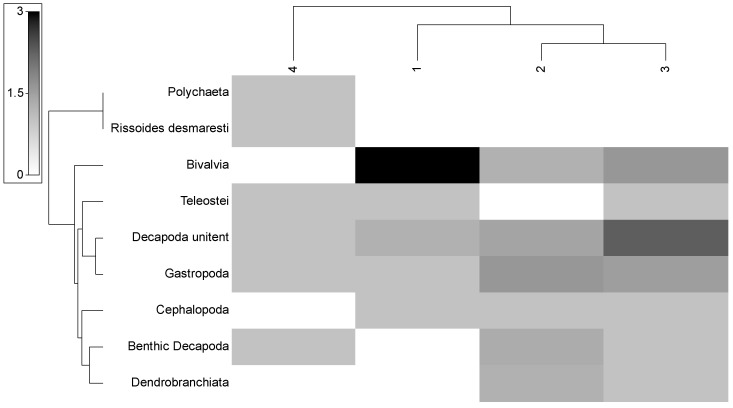
Shade plot for the mean abundance of the prey taxa and the four defined size groups of *D. maroccanus*. Shading intensity shows the relative presence of each prey in each size group. Size group 1: smaller juveniles (≤120 mm), group 2: larger juveniles (121–142 mm), group 3: smaller adults (143–164 mm), and group 4: larger adults (≥165 mm).

Due to the results of the db–RDA analysis, the following analyses were performed for four size groups: group 1—smaller juveniles (≤120 mm), group 2—larger juveniles (121–142 mm), group 3—smaller adults (143–164 mm), and group 4—larger adults (≥165 mm). The results of the cluster analysis for the similarity of the mean abundance of prey items in the four size groups are presented in Figure 5. It is obvious that size group 4 is clearly distinguished from all other size groups, while size group 1 is more discrete than the two intermediate-sized groups (2 and 3). The shade plot for the combined information on the mean abundance of the prey taxa and the four cluster size groups shows their feeding preferences in Figure 6. Polychaeta and *R. desmaresti* are exclusively presented in the diet of size group 4. Size group 1 fed primarily on Bivalvia, followed by Decapoda unidentified. Size groups 2 and 3, presenting the highest similarity, showed a more mixed diet (group 2: mainly Gastropoda, Decapoda unidentified, benthic Decapoda, and Bivalvia; group 3: mainly Decapoda unidentified, Gastropoda, and Bivalvia).

SIMPER analysis showed that size group 4 presented the highest values of dissimilarity (48.0–56.8%) with all other groups (Table 3). The lowest dissimilarity (16.6%) was found between size groups 2 and 3. The prey taxon with the highest contribution in the dissimilarity between the size groups was Bivalvia. Bivalvia and Dendrobranchiata contributed equally to the dissimilarity between groups 2 and 4, while Teleostei was between the intermediate size groups.

The diet overlap indicated the lowest values for size group 4. High overlap was defined between the two intermediate-size groups (2 and 3) and between the size groups 1 and 3 (Table 3).

The mean value of the diversity index H′ was relatively low for all size groups (Table 4) with lower values for size groups 1 and 4. No statistically significant differences were defined between the four groups (ANOVA, F-Ratio = 1.66, Df = 66, *p*-value = 0.18). Diet breadth was increased from group 1 to 4 (Table 4) indicating that the diet of the smallest individuals was characterized by the high dominance of one prey taxon, while the largest individuals fed equally on different prey taxa. The trophic level also increased from the smaller-sized to the larger-sized groups (Table 4).

## 4. Discussion

Knowledge of the feeding biology and ontogenetic changes of a species is important in population and community dynamics, which in turn is crucial for evaluating the status of the ecosystem where the species is encountered [37]. The present study investigated for the first time the diet of *D. maroccanus* in the South Aegean Sea. Additionally, the effect of body size on the feeding activity of this species was studied, an issue that has presented limited published information from other areas to date [24,25]. Although an important number of samples were examined in the current work, the number of specimens with empty stomachs was very high, a fact that may produce limits in our analyses. Indeed, although our samples included specimens of up to 233 mm TL, the larger specimen with food in the stomach was 186 mm TL. The high number of empty stomachs in the present study may be related to the sampling period, which included summer and early autumn. Bayhan et al. [24] also found a higher percentage of empty stomachs in summer, which is the species’ spawning period. It is generally known that fish reduce their feeding intensity during the reproductive period [38].

*D. maroccanus* feeding habits showed a tendency towards a more generalized feeding on Decapoda and Gastropoda, although a kind of specialization for Polychaeta and the stomatopod *R. desmaresti* was also observed. However, the majority of prey items were occasionally found in the stomach contents, reflecting the narrow feeding width of the species. This was in accordance with the results of the Shannon-Wiener diversity index, which indicated relatively low values.

In the present work, among the three examined factors of season, sex, and body size, only the latter was found to significantly affect the diet of *D. maroccanus*. Simper analysis and the shade plot showed that small individuals prey mainly on Bivalvia, while the intermediate-sized individuals (<165 mm) fed mainly on various categories of Decapoda, small Gastropoda, and small Bivalvia. The larger preys of *R. desmarsti* and Polychaeta, which require larger mouth dimensions to prey, were found only in the largest of the adults (≥165 mm). Mohdeb et al. [25] found that adult individuals target mainly Crustacea and Teleostei, as opposed to young individuals for which Crustacea were the preferred prey. However, they did not detect statistically significant differences in the diet of the species for the factors of season, sex, and body size. In contrast, Bayhan et al. [24] found that the species presented a different diet in summer compared to the other seasons. These differences may be related to the different prey availability and frequency in each study area.

The results of the diet breadth and shade plot showed that the largest size group fed equally on various prey taxa, while smaller individuals were characterized by the high dominance of a few prey categories. Similarly, Simper analysis and the estimation of the diet overlap confirmed the above-mentioned results by indicating higher dissimilarity and lower overlap, respectively, between the largest size group and the smaller ones. The estimation of the trophic level indicated higher values for the adults (>143 mm) than juveniles. Similar results for the trophic level of *D. maroccanus* have been mentioned by Mohdeb et al. [25]. The values of the trophic level confirm the carnivorous character of the species with only the larger individuals classified close to the top predators while the smaller ones presented the lowest trophic level values of this category [39].

The results of the present study showed that *D. maroccanus* is a predator that forages on benthic and demersal organisms and thereby serves as a vector of energy between benthic and pelagic ecosystems. The feeding pattern of this demersal species seems to be related to body size. Therefore, diet studies for this species should take into consideration the effect of this factor. Furthermore, the high vacuity index values have implications for a large number of samples and an extended spatiotemporal sampling design. The outcomes of the present work are useful in further understanding the species’ biology and ecology and may support the development of ecosystem-based fisheries management.

## 5. Conclusions

This study has updated our knowledge of *D. maroccanus* feeding habits and has presented for the first time information on this issue in the context of the South Aegean Sea. In this study area, *D. maroccanus* showed a narrow-width feeding strategy with a preference for Decapoda. The diversity of the prey was also relatively low. This species’ feeding habits are affected by the body size of the individuals. Therefore, large-sized prey such as Polychaeta and Stomatopoda were found only in the diet of the largest individuals, while small-sized prey like Bivalvia was mainly included in the stomach content of the smallest *D. maroccanus*. The trophic level confirmed the carnivorous character of the species with the larger individuals classified close to the top predators while the smaller presenting the lowest values of this category.

## Figures and Tables

**Table 1:** Quantitative dietary composition of *D. maroccanus*.

Prey Taxa	%N	%F	%W	Q	%IRI
ANNELIDA					
Polychaeta	0.8	1.06	0.30	0.24	0.04
MOLLUSCA					
Gastropoda	20.00	19.15	5.34	106.80	8.18
Gastropoda unident.	12.80	12.77	4.27	54.64	7.69
Chilodontaidae	0.80	1.06	0.21	0.17	0.04
Fasciolariidae	2.40	1.06	0.12	0.29	0.09
Naticidae	1.60	2.13	0.35	0.56	0.15
Pyramidellidae	2.40	2.13	0.39	0.94	0.21
Bivalvia	12.80	11.70	2.82	36.10	2.66
Bivalvia unident.	4.80	5.32	1.35	6.50	1.15
Nuculanidae	0.80	1.06	0.91	0.73	0.06
Pectinidae	7.20	5.32	0.55	3.95	1.45
Cephalopoda	4.80	6.38	18.89	90.67	5.34
ARTHROPODA-Crustacea					
Decapoda unident.	34.40	28.72	34.98	1203.30	70.32
Benthic Decapoda	15.20	18.09	10.08	153.22	6.54
Brachyura	8.80	10.64	4.13	36.34	4.85
Alpheidae	0.80	1.06	1.37	1.10	0.08
Munididae	0.80	1.06	1.22	0.97	0.08
Paguridae	4.80	5.32	3.36	16.12	1.53
Dendrobranchiata	6.40	7.45	6.65	42.58	3.43
(pelagic decapoda)					
Stomatopoda					
*Rissoides desmaresti*	1.6	2.13	11.70	18.72	1.00
CHORDATA-Teleostei	4.00	5.32	9.23	36.93	2.48
Length range (mm)	99–186				
N of individuals	67				

%N = relative abundance, %F = frequency of prey occurrence, %W = percentage weight, Q = alimentary coefficient, %IRI = index of relative importance. Length range and number of examined individuals are also presented.

**Table 2:** Results of PERMANOVA analysis with the factors affecting the abundance of prey items in *D. maroccanus* diet (statistically significant values in bold).

PERMANOVA	Abundance *p*-Value
Size	**0.012**
Sex	0.470
Season	0.146
Sex × Size	0.511
Sex × Season	0.474
Size × Season	0.140
Sex × Size × Season	0.516

**Table 3:** SIMPER analysis of the prey taxa contributing 90% to the diet of the four size groups of *D. maroccanus*. Contrib. % = contribution in abundance of the most important prey for each size group. Diet overlap values are also presented. Size group 1: smaller juveniles (≤120 mm), group 2: larger juveniles (121–142 mm), group 3: smaller adults (143–164 mm), and group 4: larger adults (≥165 mm).

Size Groups	Average Dissimilarity (%)	Prey Taxa	Contrib. (%)	Diet Overlap
1–2	40.55	Bivalvia	28.51	0.55
1–3	29.67	Bivalvia	28.36	0.65
2–3	16.58	Teleostei	34.91	0.65
1–4	54.72	Bivalvia	41.38	0.34
2–4	56.80	Bivalvia and Dendrobranchiata	15.85	0.26
3–4	48.00	Bivalvia	21.66	0.32

**Table 4:** Results of Shannon–Wiener index (H′), diet breadth (B), and trophic level (TrL) for *D. maroccanus* by size group. Size group 1: smaller juveniles (≤120 mm), group 2: larger juveniles (121–142 mm), group 3: smaller adults (143–164 mm), and group 4: larger adults (≥165 mm).

Size Group	H′	B	TrL
1	0.10 ± 0.09	0.35	3.70 ± 0.47
2	0.32 ± 0.08	0.46	3.70 ± 0.43
3	0.31 ± 0.07	0.50	4.10 ± 0.54
4	0.12 ± 0.14	0.90	4.00 ± 0.62

## Data Availability

The data presented in this study are available on request from the corresponding author.
